# Compound heterozygous c.598_612del and c.1746-20C > G *CAPN3* genotype cause autosomal recessive limb-girdle muscular dystrophy-1: a case report

**DOI:** 10.1186/s12891-021-04920-3

**Published:** 2021-12-04

**Authors:** Evelina Siavrienė, Gunda Petraitytė, Birutė Burnytė, Aušra Morkūnienė, Violeta Mikštienė, Tautvydas Rančelis, Algirdas Utkus, Vaidutis Kučinskas, Eglė Preikšaitienė

**Affiliations:** 1 Department of Human and Medical Genetics, Institute of Biomedical Sciences, Faculty of Medicine, Vilnius University, Santariskiu street 2, LT-08661 Vilnius, Lithuania; 2 Biobank of Lithuanian Population and Rare Disorders, Institute of Biomedical Sciences, Faculty of Medicine, Vilnius University, Vilnius, Lithuania

**Keywords:** *CAPN3*, LGMDR1, cDNA assay, Splicing variant, Compound heterozygosity

## Abstract

**Background:**

Autosomal recessive limb–girdle muscular dystrophy-1 (LGMDR1), also known as calpainopathy, is a genetically heterogeneous disorder characterised by progression of muscle weakness. Homozygous or compound heterozygous variants in the *CAPN3* gene are known genetic causes of this condition. The aim of this study was to confirm the molecular consequences of the *CAPN3* variant NG_008660.1(NM_000070.3):c.1746-20C > G of an individual with suspected LGMDR1 by extensive complementary DNA (cDNA) analysis.

**Case presentation:**

In the present study, we report on a male with proximal muscular weakness in his lower limbs. Compound heterozygous NM_000070.3:c.598_612del and NG_008660.1(NM_000070.3):c.1746-20C > G genotype was detected on the *CAPN3* gene by targeted next-generation sequencing (NGS). To confirm the pathogenicity of the variant c.1746-20C > G, we conducted genetic analysis based on Sanger sequencing of the proband’s cDNA sample. The results revealed that this splicing variant disrupts the original 3′ splice site on intron 13, thus leading to the skipping of the DNA fragment involving exon 14 and possibly exon 15. However, the lack of exon 15 in the *CAPN3* isoforms present in a blood sample was explained by cell-specific alternative splicing rather than an aberrant splicing mechanism. In silico the c.1746-20C > G splicing variant consequently resulted in frameshift and formation of a premature termination codon (NP_000061.1:p.(Glu582Aspfs*62)).

**Conclusions:**

Based on the results of our study and the literature we reviewed, both c.598_612del and c.1746-20C > G variants are pathogenic and together cause LGMDR1. Therefore, extensive mRNA and/or cDNA analysis of splicing variants is critical to understand the pathogenesis of the disease.

**Supplementary Information:**

The online version contains supplementary material available at 10.1186/s12891-021-04920-3.

## Background

Limb–girdle muscular dystrophies (LGMD) are a group of genetically heterogeneous disorders characterised by a wide spectrum of clinical features and different rates of progression. More than 30 different genetic subtypes of LGMD have been identified. Autosomal recessive limb–girdle muscular dystrophy-1 (LGMDR1; MIM #253600; ORPHA #267), which was formerly referred to as LGMD2A, is the most prevalent form of LGMD in many countries [1, 2].

Although the underlying pathological mechanisms involved in LGMDR1 are not well known, studies of *Capn3* knockdown mice identified a human calpainopathy-like phenotype, thus indicating that this disease is caused by defects in the *CAPN3* gene (MIM #114240), which is located on chromosome 15q15 [3]. A number of different studies have demonstrated that the homozygous or compound heterozygous genotype of *CAPN3* lead to the pathogenesis of LGMDR1. Meanwhile, the heterozygous *CAPN3* variants have been reported to cause autosomal dominant limb–girdle muscular dystrophy-4 (LGMDD4; MIM #618129; ORPHA # 102014), which has a later onset and milder phenotype than LGMDR1 [2, 4, 5]. Besides DNA sequence variants in the *CAPN3* gene, it has been suggested that other genetic and environmental factors modulate the clinical phenotype of LGMDR1, because variable degrees of muscle involvement and disease progression have been reported in individuals with the same genetic variant [6].

The Human Gene Mutation Database (HGMD) and Leiden Open Variation Database (LOVD) report more than 400 pathogenic DNA sequence variants of the *CAPN3* gene, the majority of which are missense or nonsense variants [7, 8]. The splicing variants accounting for about 15% of total variants are also a common cause of LGMDR1. Since the consequences of splicing variants on mRNA transcripts have been mostly inferred by in silico analysis, it is conceivable that the frequency of these disease-causing variants could be higher after experimental complementary DNA (cDNA) analysis. In order to accurately assess the pathogenicity of splicing variants at the mRNA level, detailed molecular and/or functional analysis is required [6, 9]. Therefore, this research strategy has been used in this study to confirm the molecular consequences of the variant NG_008660.1(NM_000070.3):c.1746-20C > G of the *CAPN3* gene of an individual with LGMDR1.

## Case presentation

The proband, a 43-year-old Lithuanian man, with proximal muscular weakness in his lower limbs, presented initially at age 17 with walking difficulties. Five years later he developed weakness in his shoulder girdle. A neurological examination at the age of 37 revealed atrophy of his shoulder girdle, wasting of biceps and triceps, thinning of thighs, pseudo hypertrophy of both calves, and winged scapulae. Gower’s sign was positive. Muscle strength was 4/5 (MRC-scale) at both shoulders, 4/5 at both elbows, 4/5 at both wrists, 3/5 at both hip joints, 3/5 at both knees, and 2/5 at both ankles. His facial muscles were not involved. His creatine kinase (CK) level was 3451 U/L (ref. range < 195 U/L). Needle electromyography showed chronic myopathic changes. The proband did not have any cardiac or pulmonary involvement. Family history was unremarkable. The clinical phenotype, age of presentation, and course of the disease were consistent with a diagnosis of LGMDR1 [10].

With a clinical suspicion of muscular dystrophy or myopathy, targeted amplicon sequencing was performed using next-generation sequencing (NGS). Genomic DNA (gDNA) was extracted from peripheral venous blood samples of our proband and his family members following the phenol-chloroform-isoamyl alcohol protocol [11]. Targeted amplicon sequencing was done for this individual using the gene panel for neuromuscular disorders on an Ion Torrent (Ion Personal Genome Machine; Thermo Fisher Scientific, USA). Multiplex primer pools were designed using Ion AmpliSeq Designer software (Thermo Fisher Scientific, USA). This custom gene panel consisted of 297 genes (Table S2) and covered 99.96% of the exonic region, including the flanking exon–intron boundary regions. Enrichment of exonic sequences was performed with an Ion AmpliSeq Library Kit 2.0 (Thermo Fisher Scientific, USA) and sequenced on an Ion PGM (Thermo Fisher Scientific, USA) using 318 Chip (Thermo Fisher Scientific, USA) according to the manufacturer’s protocol. Data analysis was performed using Ion Torrent software (Thermo Fisher Scientific, USA). After the analysis pipeline was processed, 1194 genome variants were identified from obtained sequence data. ANNOVAR [12] was used to annotate and prioritize DNA sequence variants of the *CAPN3* gene. After filtering methods were applied, only one non-frameshift deletion (rs727503837) of the *CAPN3* gene has matched with proband’s phenotype. This deletion has been previously confirmed as pathogenic in a few individuals with LGMDR1 (PMID #9452114 [13], #25135358 [14], #18854869 [15], #16141003 [16]) as well as ClinVar [17] database. Therefore, this deletion was the main candidate for the proband’s phenotype. Based on the clinical phenotype, age of presentation, and course of the disease, the autosomal recessive (R1) subtype of the LGMD has been suspected. For this reason, the hypothesis of compound heterozygosity has been raised. The intronic variant (rs201892814) in the *CAPN3* gene, which has low frequency in population and has damaging value by RegSNPs-intron tool [18], was selected as another possible candidate. The classification of these genetic variants for the proband’s phenotype was assessed using criteria outlined by the American College of Medical Genetics (ACMG) [19]. The c.598_612del variant was classified as “pathogenic”, according to such criteria as PS4, PM1, PM2, PM4, PP1, PP3. Based on the PS3, PM2, PM4, PP3 criteria, the second c.1746-20C > G variant was classified as “likely pathogenic”.

Confirmation of *CAPN3* variants identified by NGS and familial segregation analysis were performed through Sanger sequencing using BigDye Terminator v.3.1 Cycle Sequencing Kit and ABI3130xl automated sequencer (Life Technologies, USA). Polymerase chain reaction (PCR) of *CAPN3* exon 4 and exon 14 as well as further Sanger sequencing was performed according to the manufacturer’s protocol on proband’s and his parents’ samples. The relative exons and flanking intron regions were amplified using primers (Table S1) designed by Primer3 [20]. Sanger sequencing results were analysed with Sequence Analysis v.5.1 (Thermo Fisher Scientific, USA) and Chromas v.2.4.4 (Technelysium Pty Ltd., Australia) software. The resulting sequence was aligned with the reference sequence of the *CAPN3* gene (NCBI: NG_008660.1).

To confirm the pathogenicity of the detected splice site variant, genetic analysis of proband’s cDNA sample was performed. Total RNA of the proband was extracted from the whole blood using Tempus™ Blood RNA Tube and Tempus™ Spin RNA Isolation Kit (Thermo Fisher Scientific, USA) according to the optimized manufacturers’ protocols. Complementary DNA (cDNA) was synthesized from total RNA using a High Capacity RNA-to-cDNA Kit (Thermo Fisher Scientific, USA) following manufacturer’s protocol. PCR of cDNA sequence flanking *CAPN3* splicing variant was performed using specific primers (Table S1) designed with Primer Blast [21]. Proband’s PCR products were sequenced via Sanger sequencing technique and aligned with the reference sequence of the *CAPN3* gene (NCBI: NM_000070.3) as indicated above.

The pathogenicity of identified DNA sequence variants was evaluated by a review of scientific literature and analysis of databases, such as HGMD [7], LOVD [8], 1000 genomes [22], dbSNP [23], ClinVar [17], OMIM [24], Orphanet [25], Ensembl [26], and gnomAD browser [27]. MutationTaster 2 [28], Human Splicing Finder [29], and regSNPs splicing [30] tools were used for predicting splice site alterations. Sequences of evolutionary distinct species were obtained from the Ensembl genome browser [26], while a sequence alignment was produced using ClustalO tool [31]. Possible splice site variant’s effect on the CAPN3 protein (UniProtKB: P20807) was predicted using different tools and databases, e.g., UniProt [32], ExPASy Bioinformatics Resource Portal [32], and Pfam 32.0 database [33].

**Fig. 1:**
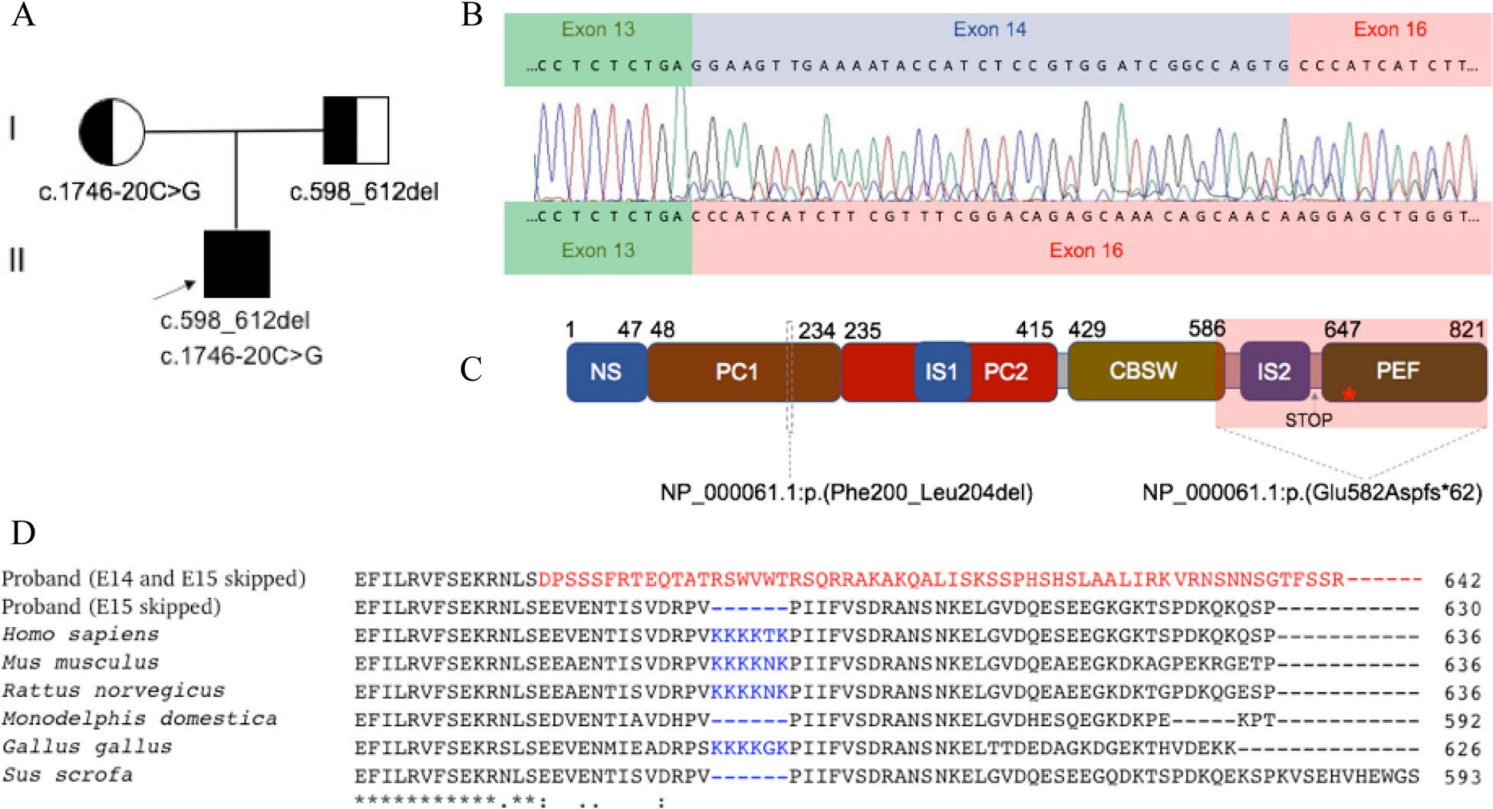
**A** Segregation of compound heterozygous variants c.598_612del and c.1746-20C > G of *CAPN3* in the family; **B** Representative Sanger sequencing electropherogram of the proband’s cDNA sample showed two different transcripts corresponding to the isoform lacking exon 15 and the isoform lacking both exon 14 and exon 15; **C** A schematic representation of the CAPN3 protein, which is 821 amino acids in length, with the approximate position of the premature termination (STOP) codon in relation to the last exon-exon junction (*) and the arrangement of the main domains: N-terminal addition sequence (NS), two protease core domains (PC1 and PC2), insertion sequence 1 (IS1), calpain-type β-sandwich domain (CBSW), insertion sequence 2 (IS2), and penta E-F hand domain (PEF) [50]; **D** A comparative sequence alignment produced by ClustalO of the CAPN3 protein across six evolutionarily distant species. Frameshift of 62 new amino acids and therefore a truncated sequence due to lack of both exon 14 (E14) and exon 15 (E15) is highlighted in red. Blood-specific alternative splicing of exon 15 (E15), which encodes six amino acids, is highlighted in blue

After targeted NGS and in silico analysis, compound heterozygous NM_000070.3:c.598_612del and NG_008660.1(NM_000070.3):c.1746-20C > G *CAPN3* genotype were detected. Sanger sequencing confirmed the segregation of these variants in the family: the father was determined to be a heterozygous carrier of the c.598_612del variant, while the mother was heterozygous for the c.1746-20C > G variant (Fig. 1A). In silico analysis revealed that the c.598_612del variant has been reported as an intragenic deletion (rs727503837) that leads to the loss of five amino acids (NP_000061.1:p.(Phe200_Leu204del)) at the protein level (Fig. 1C). Even though the second variant, c.1746-20C > G (rs201892814), with the minor allele frequency (MAF) lower than 0.01, is also rare, the interpretation of its pathogenicity based on ClinVar [17] and LOVD data [8] is still uncertain. A variant effect prediction method, MutationTaster 2 [28], predicted the variant to be benign. However, Human Splicing Finder [29] and regSNPs [30] predicted that this DNA sequence variant would most probably affect splicing of precursor messenger RNA (pre-mRNA) by activation of an intronic cryptic acceptor site. Sequence alignment of the CAPN3 protein across six evolutionarily distant species revealed that the region flanking the splicing variant is highly conserved (Fig. 1D). Sanger sequencing results showed two different transcripts corresponding to the isoform without exon 15 and the isoform without both exon 14 and exon 15 (Fig. 1B). Further computational analysis demonstrated that the isoform without exon 15 leads to the loss of six amino acids (NP_000061.1:p.(Lys595_Lys600del)), while the isoform without both exon 14 and 15 results in a translational frameshift of 62 amino acids and formation of a premature termination codon (NP_000061.1:p.(Glu582Aspfs*62)) (Fig. 1C).

## Discussion

DNA sequence variants altering pre-mRNA splicing account for at least 15% of disease-causing variants [34]. These variants affect pre-mRNA processing through exon skipping, intron inclusion, cryptic splicing, leaky splicing, or even introduction of pseudo-exons into the processed mRNA. Since these genetic variants have different consequences, they are especially difficult to predict by in silico analysis. In order to accurately assess the effects of splicing variants at the mRNA level, experimental mRNA or cDNA analysis is necessary [9, 35, 36].

In this study, we present a characterisation of the *CAPN3* splicing variant (NG_008660.1(NM_000070.3):c.1746-20C > G), for which there is conflicting data. This variant was earlier identified in several unrelated individuals with LGMDR1 [2, 37–39]. However, the consequences of c.1746-20C > G are mostly unsupported by experimental data; only a few studies have reported results based on cDNA and/or protein analysis. After performing cDNA and Western blot analysis, Krahn et al. (2007) reported no changes in *CAPN3* mRNA transcripts but did find reduced protein expression [38]. In a similar study, Nascimbeni et al. (2010) reported that the c.1746-20C > G variant altered both mRNA splicing and protein expression [2]. In independent parallel study Macias et al. (2021) researched the *CAPN3* variants in non-coding regions or potential regulatory sequences in individuals with the LGMDR1 phenotype. The compound heterozygous c.598_612del and c.1746-20C > G *CAPN3* genotype was also detected in four unrelated individuals. Similarly to our proband, they all presented uniformly with mature adult disease onset and no contractures. However, in our proband lower-limb weakness was predominant, while two individuals presented by Macias et al. (2021) had prevailing upper-limb weakness. Even though the reduction of the CAPN3 protein amount in most Western blot samples was detected, analysis of mRNA transcripts was not performed in this parallel study [40]. Therefore, the molecular consequences of the c.1746-20C > G splicing variant at cDNA level are still unknown.

**Fig. 2:**
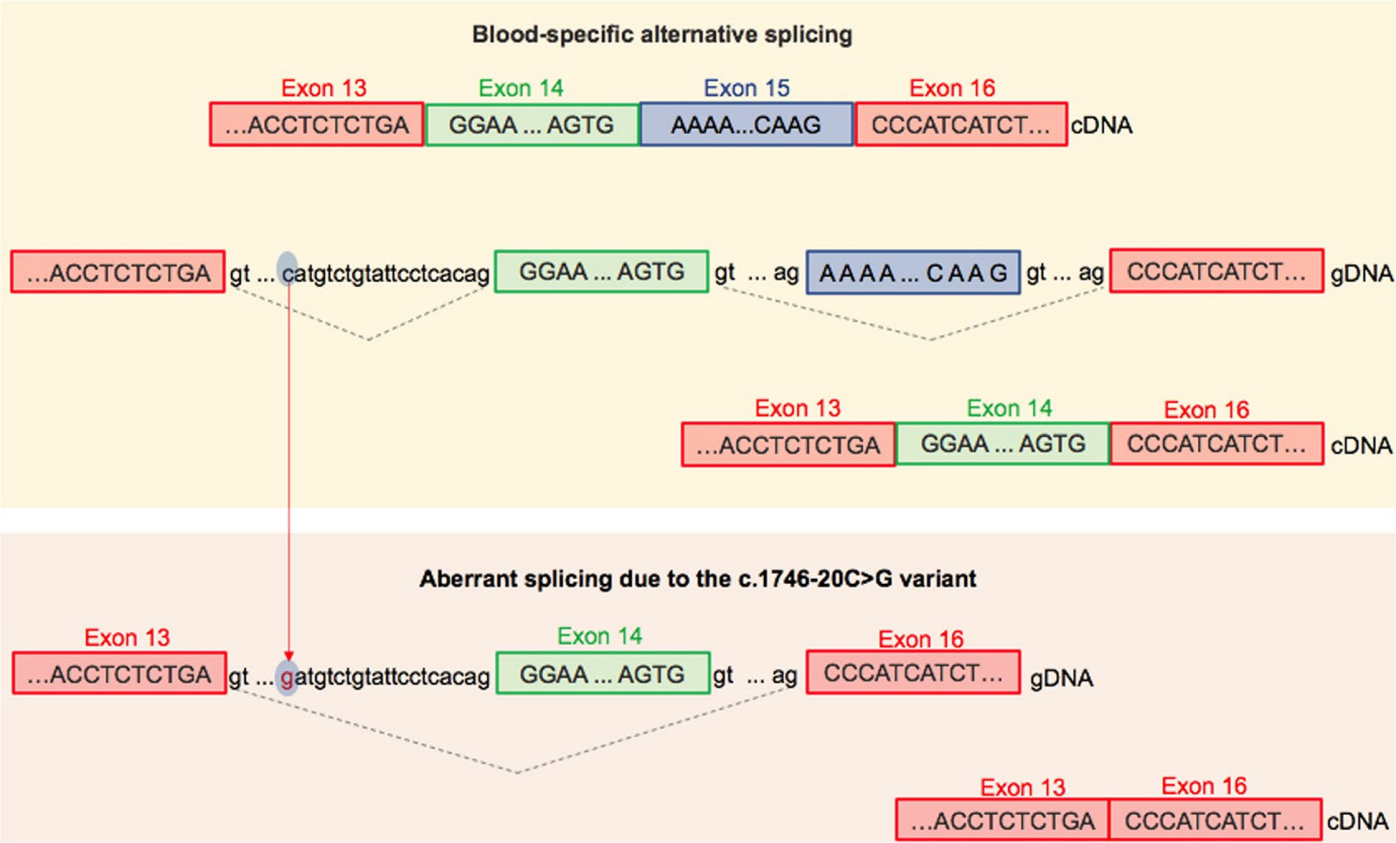
Schematic representation of *CAPN3* reference and mutant sequence with the splicing variant c.1746-20C > G

Deep intronic variants, including c.1746-20C > G, which is located twenty nucleotides upstream of the 3′ acceptor site, usually create new splice sites resulting in the inclusion of cryptic exons. In the study done by Nascimbeni et al. (2010), the c.1746-20C > G splicing variant resulted in the exonisation of all or part of intron 13 in six unrelated individuals with LGMDR1. However, Sanger sequencing of the proband’s cDNA sample in the present study revealed that this variant disrupts the original 3′ splice site in intron 13, thus leading to skipping of exon 14 and possibly exon 15. Two different transcripts corresponding to the isoform lacking exon 15 and the isoform lacking both exon 14 and exon 15 have been identified with possibly lower quantity of the latter in the cDNA sample (Fig. 1B). The presence of different mRNA transcripts in the blood sample of our proband could be explained as different consequences of a mutant splicing mechanism or blood-specific alternative splicing. As demonstrated in the study performed by Nascimbeni et al. (2010), the same pathogenic splicing variant can result in two or more aberrant transcripts in a certain tissue [2]. On the other hand, pre-mRNA splicing is a normal cellular process contributing to the production of functional protein and ensuring protein diversity in the cell. For instance, the study of the *NF1*-coding region of two non-affected individuals showed 46 different transcripts of this gene [9, 41]. Also, different transcripts can be expressed in diverse tissues or cell types, thus ensuring specific biological functions [42]. In an investigation of 26 individuals with LGMDR1, Blázquez et al. (2008) identified four different *CAPN3* mRNA transcripts in white blood cells (WBCs): an isoform lacking exon 15, an isoform lacking exons 15 and 16, an isoform lacking exons 6 and 15, and an isoform without these three exons. Moreover, the comparative analysis of peripheral blood and muscle samples showed that the c.1782 + 1072G > C splicing variant results in the insertion of 100 bp of intron 14 between exons 14 and 16 in WBCs and between exons 14 and 15 in the muscle sample [5]. Based on these findings, the absence of exon 15 in all the isoforms in individuals’ peripheral blood samples could be explained by the alternative splicing isoform rather than the mutant isoform. Therefore, the c.1746-20C > G splicing variant in the proband’s cDNA sample is most likely to result in the loss of only exon 14, while the loss of exon 15 is supposed to be caused by the blood-specific alternative splicing (Fig. 2B).

In silico, the *CAPN3* c.1746-20C > G splicing variant causes a translational frameshift and formation of a premature termination codon, thus resulting either in protein truncation or mRNA degradation due to nonsense-mediated decay (NMD). During this cellular process, the degradation of mRNAs that harbour a premature termination codon at least 200 nucleotides downstream of the start codon and 50–55 nucleotides upstream of the last exon–exon junction can be induced [43–45]. The premature termination codon induced by the c.1746-20C > G splicing variant of the *CAPN3* gene conforms to the conditions for NMD (Fig. 1C), and therefore the mRNA degradation process could be initiated. However, the presence of both wild type and altered transcript in our study indicates that these transcripts do not undergo full NMD but may also result in the production of the truncated protein. To confirm this assumption, more detailed analysis (e.g., Western blot) at the protein level could be suggested. Nevertheless, both partial reduction of mRNA transcripts and truncated protein due to the *CAPN3* splicing variant would be deleterious and contribute to LGMDR1 pathogenesis.

LGMDR1 was the first human disease found to be caused by alterations of the enzyme calpain. Calpain-3 (UniProtKB #P20807), which is encoded by the *CAPN3* gene, belongs to the calpain superfamily of Ca^2+^-regulated non-lysosomal cysteine proteases and are found in almost all eukaryotes [3]. According to the GTEx Portal [46], *CAPN3* is expressed in almost all human tissues. The product of *CAPN3* therefore has essential functions in many cellular processes, including cell differentiation and motility, apoptosis, and regulation of the cell cycle and signal transduction system. The conventional calpains (CAPN1, CAPN2, and CAPN3) are structurally similar and share four domains: two protease core domains (PC1 and PC2), a calpain-type β-sandwich domain (CBSW), and a penta E-F hand domain (PEF), which are critical for Ca^2+^-dependent protein activation. Differently from other calpain family members, CAPN3 also has three unique regions: N-terminal addition sequence (NS), insertion sequence 1 (IS1), and insertion sequence 2 (IS2) (Fig. 1C). Due to these unique regions, CAPN3 has additional properties: Na^+^-dependent protein activation and extremely fast autodegradation. Moreover, previous studies have shown that the N2A region of titin, which is encoded by *TTN* (MIM #188840), binds to the IS2 region and stabilises CAPN3 by preventing its autodegradation. The IS2 region, which is partly encoded by exon 15 of *CAPN3*, therefore plays an important role in regulation of gene expression [47–49]. Based on GTEx data [46], the highest expression of both *CAPN3* and *TTN* is reported in skeletal muscles, while expression of these genes in whole blood samples is minimal. Since their functions are considered non-essential in whole blood samples and there is no need to stabilise the CAPN3 protein, the *CAPN3* mRNA transcript without exon 15 could be explained by cell-specific alternative splicing occurred during evolutionary processes. Furthermore, the sequence alignment of the CAPN3 protein across six evolutionarily distant species revealed that exon 15, encoding only six amino acids, is also alternatively spliced in the cDNA sequence of opossums (*Monodelphis Domestica*) and pigs (*Suc Scrofa*). Contrarily, the region spanning the splicing variant c.1746-20C > G is highly conserved in all organisms that have been analysed, thus suggesting that this region is fundamentally important (Fig. 1D).

The compound heterozygous c.598_612del and c.1746-20C > G *CAPN3* genotype was predicted by computational analysis to affect the proband’s functional protein domains. At the protein level, the c.598_612del variant detected in exon 4 was predicted to result in a loss of five amino acids (NP_000061.1:p.(Phe200_Leu204del) in the PC1 domain. Meanwhile, a variant disrupting the original 3′ splice site in intron 13 was predicted to result in either partial degradation of mRNA transcripts or a translational frameshift and formation of premature termination codon. In the case of a protein truncation (NP_000061.1:p.(Glu582Aspfs*62), CAPN3 lacks IS2, PEF, and part of the CBSW domain due to the c.1746-20C > G splicing variant (Fig. 1C). Based on the results of our study and the literature we reviewed, both DNA sequence variants are pathogenic and cause LGMDR1.

## Limitations

The predicted lower expression of the CAPN3 protein has not been confirmed by Western blotting, because of the unavailability of proband’s cell culture.

## Conclusions

In this study we report a compound heterozygous c.598_612del and c.1746-20C > G *CAPN3* genotype leading to LGMDR1. Moreover, by confirming the consequences of the splicing variant c.1746-20C > G at the mRNA level, we provide additional evidence that extensive mRNA and/or cDNA analysis is highly informative for the evaluation of these DNA sequence variants and the relationship between genotype and phenotype. This research strategy could be suggested in the routine practice of medical genetics.

## Supplementary Information

**Additional file 1:** **Supplementary Table 1.** The conditions for PCR amplification of the *CAPN3* gene in gDNA and cDNA samples.

**Additional file 2: Supplementary Table 2.** The list of genes that are included in the Ion AmpliSeq™ On-Demand panel for targeted sequencing.

## Data Availability

The main data generated and analysed during this study are included in this article and its supplementary information file. Any additional information is available from the authors upon request.

## Abbreviations

*CAPN3* gene: Calpain 3
CBSW: Calpain-type β-sandwich domain
cDNA: Complementary DNA
CK: Creatine kinase
ClustalO: Clustal Omega sequence alignment program
dbSNP: The Single Nucleotide Polymorphism Database
gDNA: Genomic DNA
gnomAD: The Genome Aggregation Database
HGMD: The Human Gene Mutation Database
IS1: Insertion sequence 1
IS2: Insertion sequence 2
LGMD: Limb–girdle muscular dystrophies
LGMDD4: Autosomal dominant limb–girdle muscular dystrophy-4
LGMDR1 or LGMD2A: Autosomal recessive limb–girdle muscular dystrophy-1
LOVD: Leiden Open Variation Database
MAF: Minor allele frequency
*NF1* gene: Neurofibromin 1
NGS: Next-generation sequencing
NMD: Nonsense-mediated decay
NS: N-terminal addition sequence
OMIM: Online Mendelian Inheritance in Man
PC1: Protease core domain 1
PC2: Protease core domain 2
PCR: Polymerase chain reaction
PEF: Penta E-F hand domain
pre-mRNA: Precursor messenger RNA
*TTN* gene: Titin
WBCs: White blood cells
