# Supplementary material for: Compound heterozygous c.598_612del and c.1746-20C > G CAPN3 genotype cause autosomal recessive limb-girdle muscular dystrophy-1: a case report
Source: BMC Musculoskelet Disord. 2021 Dec 4;22:1020. doi: 10.1186/s12891-021-04920-3 (PMC8645139; doi:10.1186/s12891-021-04920-3)
Supplement: Supplementary file 1 — Additional file 1: Supplementary Table 1. The conditions for PCR amplification of the CAPN3 gene in gDNA and cDNA samples. [file 12891_2021_4920_MOESM1_ESM.docx]

**Supplementary Table 1**. The conditions for PCR amplification of the *CAPN3* gene in gDNA and cDNA samples.

| Gene  (RefSeq transcript) | Sample | Primers (5’ 🡪 3’) | Location | Amplicon length |
| --- | --- | --- | --- | --- |
| *CAPN3* (NG_008660.1) | gDNA | **F:** TCTCAAAAAGCACCCAGTCC | Intron 3 | 419 bp |
|  |  | **R:** GCCTCTTCCTGTGAGTGAG | Intron 4 |  |
|  |  | **F:** GGGGTTCTCTAGAGGCTGGT | Intron 13 | 244 bp |
|  |  | **R:** ATTCTCCCCTGCACCATCT | Intron 14 |  |
| *CAPN3*  (NM_000070.3) | cDNA | **F:** GCTTCGCCATCTACGAGGTT | 11–12 exon junction | 380 bp |
|  |  | **R:** GCTTGTTTTGCCTTTGCCCT | Exon 16 |  |

F – forward primer; R – reverse primer
