# Supplementary material for: Compound heterozygous c.598_612del and c.1746-20C > G CAPN3 genotype cause autosomal recessive limb-girdle muscular dystrophy-1: a case report
Source: BMC Musculoskelet Disord. 2021 Dec 4;22:1020. doi: 10.1186/s12891-021-04920-3 (PMC8645139; doi:10.1186/s12891-021-04920-3)
Supplement: Supplementary file 2 — Additional file 2: Supplementary Table 2. The list of genes that are included in the Ion AmpliSeq™ On-Demand panel for targeted sequencing. [file 12891_2021_4920_MOESM2_ESM.docx]

**Supplementary Table 2.** The list of genes that are included in the Ion AmpliSeq™ On-Demand panel for targeted sequencing.

| *A2ML1* | *CHKB* | *FTL* | *LAMP2* | *PLA2G6* | *SLC25A20* |
| --- | --- | --- | --- | --- | --- |
| *AARS* | *CYP27A1* | *G6PC* | *LARGE* | *PLP1* | *SLC2A1* |
| *ABHD5* | *CYP2U1* | *GAA* | *LDB3* | *PMP22* | *SLC33A1* |
| *ACAD9* | *CYP7B1* | *GAD1* | *LDHA* | *PNKD* | *SMCHD1* |
| *ACADL* | *CIZ1* | *GAN* | *LITAF* | *PNPLA2* | *SOS1* |
| *ACADM* | *CLCN1* | *GARS* | *LMNA* | *POLG* | *SOX10* |
| *ACADS* | *CLN3* | *GBA2* | *LPIN1* | *POLG2* | *SPAST* |
| *ACADVL* | *CNTN1* | *GBE1* | *LRSAM1* | *POMGNT1* | *SPG11* |
| *ACY1* | *COL12A1* | *GCDH* | *MAP2K1* | *POMGNT2* | *SPG20* |
| *ACTB* | *COL4A1* | *GCH1* | *MAP2K2* | *POMT1* | *SPG21* |
| *ACVR1* | *COL6A3* | *GDAP1* | *MARS* | *PRKAG2* | *SPG7* |
| *ADAR* | *CPT1C* | *GYG1* | *MATR3* | *PRKRA* | *SPR* |
| *ADCY5* | *CPT2* | *GYS1* | *MEGF10* | *PRPS1* | *SPRED1* |
| *AGL* | *CRYAB* | *GJB1* | *MFN2* | *PRRT2* | *SPTLC1* |
| *AIFM1* | *CSF1R* | *GJB3* | *MYF6* | *PRX* | *SPTLC2* |
| *AKT3* | *DAG1* | *GNB4* | *MYH2* | *PTPN11* | *STAMBP* |
| *ALDH18A1* | *DCAF8* | *GNE* | *MYH7* | *PUS1* | *STIM1* |
| *ALS2* | *DCTN1* | *HACE1* | *MYOT* | *RAB7A* | *SUCLA2* |
| *AMPD1* | *DDHD1* | *HADH* | *MLC1* | *RAF1* | *SUN2* |
| *AMPD2* | *DDHD2* | *HADHA* | *MME* | *RASA1* | *TAF1* |
| *ANO3* | *DES* | *HADHB* | *MPV17* | *RASA2* | *TAZ* |
| *ANO5* | *DHTKD1* | *HARS* | *MPZ* | *RBCK1* | *TCAP* |
| *AP4B1* | *DYNC1H1* | *HEXA* | *MSTN* | *REEP1* | *TECPR2* |
| *AP4E1* | *DYSF* | *HINT1* | *MTM1* | *RYR1* | *TFG* |
| *AP4M1* | *DMD* | *HK1* | *MTMR14* | *RIT1* | *TH* |
| *AP5Z1* | *DNA2* | *HOXD10* | *MTMR2* | *RRM2B* | *THAP1* |
| *ARHGEF10* | *DNAJB6* | *HPCA* | *NAGLU* | *RTN2* | *TIA1* |
| *ARSA* | *DNM2* | *HRAS* | *NEFL* | *SBF1* | *TIMM8A* |
| *ATL1* | *DNMT1* | *HSPB1* | *NF1* | *SBF2* | *TK2* |
| *ATM* | *DPM1* | *HSPB8* | *NGF* | *SCN10A* | *TMEM43* |
| *ATP1A3* | *DPM2* | *HSPD1* | *NIPA1* | *SCN11A* | *TMEM5* |
| *ATP2A1* | *DPM3* | *YARS* | *NPC1* | *SCN4A* | *TNPO3* |
| *ATP2B4* | *DRP2* | *YARS2* | *NPC2* | *SCN9A* | *TOR1A* |
| *ATP7A* | *DST* | *IGHMBP2* | *NRAS* | *SEPN1* | *TPM2* |
| *ATP7B* | *EGR2* | *IKBKAP* | *NTRK1* | *SEPT9* | *TPM3* |
| *AUH* | *EMD* | *INF2* | *OPA1* | *SGCA* | *TRAPPC11* |
| *B3GALNT2* | *ENTPD1* | *ISCU* | *PABPN1* | *SGCB* | *TRIM2* |
| *B4GALNT1* | *ERLIN2* | *ITGA7* | *PANK2* | *SGCD* | *TRIM32* |
| *B4GAT1* | *ETFA* | *KANK1* | *PARK2* | *SGCE* | *TRPV4* |
| *BAG3* | *ETFB* | *KBTBD13* | *PDHA1* | *SGCG* | *TTR* |
| *BCAP31* | *ETFDH* | *KCNE3* | *PDK3* | *SH3TC2* | *VCP* |
| *BRAF* | *FA2H* | *KCNJ2* | *PFKM* | *SHOC2* | *VMA21* |
| *BSCL2* | *FBLN5* | *KCNJ5* | *PGAM2* | *SIL1* | *VPS13A* |
| *C10orf2* | *FHL1* | *KCNMA1* | *PGK1* | *SYNE1* | *VPS37A* |
| *C12orf65* | *FHL2* | *KIAA0196* | *PGM1* | *SYNE2* | *WNK1* |
| *C19orf12* | *FIG4* | *KIF1B* | *PHKA1* | *SLC12A6* | *ZFYVE26* |
| *CACNA1S* | *FKBP14* | *KLHL9* | *PHKG2* | *SLC16A1* | *ZFYVE27* |
| *CAPN3* | *FKRP* | *KRAS* | *PYGM* | *SLC19A3* |  |
| *CAV3* | *FKTN* | *L1CAM* | *PIK3CA* | *SLC22A5* |  |
| *CBL* | *FLNC* | *LAMA2* | *PIK3R2* | *SLC25A15* |  |
